# Evolution of neurodegeneration in patients with normal pressure hydrocephalus: a monocentric follow up study

**DOI:** 10.1186/s42466-023-00272-6

**Published:** 2023-09-07

**Authors:** Leonard L. Klemke, Katharina Müller-Schmitz, Aschwin Kolman, Rüdiger J. Seitz

**Affiliations:** Centre for Neurology and Neuropsychiatry, LVR-Klinikum Düsseldorf, Medical Faculty, Heinrich-Heine-University Düsseldorf, Bergische Landstraße 2, 40629 Düsseldorf, Germany

**Keywords:** Normal pressure hydrocephalus, Neurodegeneration, Alzheimer's dementia

## Abstract

**Background:**

The aim of this study was to examine in patients with idiopathic and neurodegenerative normal pressure hydrocephalus (NPH) if motor and cognitive performance as well as changes in biomarkers in cerebrospinal fluid (CSF) evolve differently.

**Methods:**

41 patients with a typical clinical and MR-/CT-morphological presentation of NPH divided into an Alzheimer-negative (AD–, *n* = 25) and an Alzheimer-positive (AD+, *n* = 16) group according to neurodegenerative biomarkers (S100 protein, neuron-specific enolase, β-amyloid 1–42, Tau protein, phospho-Tau, protein-level and CSF pressure) in CSF. Follow-up of cognitive and gait functions before and after a spinal tap of 40–50 ml CSF of up to 49 months. Clinical, motor, neuropsychological and CSF biomarkers were analyzed using a repeated multifactorial analysis of variance (ANOVA) with post-hoc testing.

**Results:**

Gait and neuropsychological performance and CSF biomarkers evolved differently between the AD− and AD+ patients. In particular, the AD+ patients benefited from the spinal tap regarding short-term memory. In contrast, gait parameters worsened over time in the AD+ patients, although they showed a relevant improvement after the first tap.

**Conclusions:**

The results substantiate the recently reported association between a tap-responsive NPH and CSF changes of Alzheimer disease. Furthermore, they suggest that the AD changes in CSF manifest in an age-related fashion in AD− patients presenting with NPH.

**Supplementary Information:**

The online version contains supplementary material available at 10.1186/s42466-023-00272-6.

## Introduction

Normal pressure hydrocephalus (NPH), first described by Hakim (1922–2011) and Adams (1922–2008) as an “occult hydrocephalus with ‘normal’ cerebrospinal fluid (CSF) pressure” in 1964, is a disorder of the elderly [1]. Relkin et al. [2] summarized its clinical characterization by progressive gait impairment, cognitive disturbances and urinary urgency (Hakim-Triad). The gait disturbance is most commonly described as wide-based, shuffling gait or as gait apraxia [3]. The neuropsychological deficits include fronto-cortical dysfunction, a.e impaired psychomotor speed, impaired executive function, decreased attention and behavioral changes [4]. In the elderly the estimated prevalence of probable NPH is approximately 0.2% in those aged 70–79 years and 5.9% in those aged 80 years and older [5].

Less than 60% of suspected NPH patients present with the typical triad and the clinical appearance can vary among the patients [6]. There is no causal treatment so far but CSF drainage by a spinal tap or ventricular shunting is an effective and possible long lasting therapy for NPH patients [7, 8]. Ventricular shunting is widely considered when removal of CSF improves gait, neuropsychological function and urinary control [9]. Many of the elderly patients with NPH have additional conditions that impair the beneficial effect of shunting [10]. As the shunt implantation, however, can lead to perioperative complications (e.g. infections, shunt dysfunction) and increased risk of death, the shunt-intervention must be well gauged [11, 12].

The variable response to the spinal tap over subsequent years and after shunting provoked questioning idiopathic NPH (iNPH) as a clinical entity [13, 14]. It was argued that patients with progressive dementia, such as Alzheimer disease, Parkinson’s syndrome, or subcortical arteriosclerotic leukoencephalopathy, might present with similar symptoms and even show transient clinical improvement upon a spinal tap. In fact, in a cross-sectional study it was found that a considerable proportion of patients with NPH show a CSF biomarker signature typical for Alzheimer disease (AD+) [15]. Unexpectedly, however, these patients were found to improve upon a spinal tap in contrast to patients with NPH and no such CSF changes (AD−). These findings prompted us to investigate if patients with NPH and AD+ fared differently to patients with NPH and AD− in a longitudinal follow-up investigation using standardized neuropsychological testing and validated quantitative investigator-independent tests of gait and mobility [16]. To the best of our knowledge there are no prospective studies on changes in CSF biomarkers and motor and cognitive functions outcome in longitudinal time-trends comparing AD+ and AD− patients suffering from NPH.

It ought to be stated explicitly that the patients with an improvement after a spinal tap concerning cognitive abilities, gait and urine incontinence were considered as candidates for ventricular shunt implantation. However, before this surgical procedure was recommended to them in our centre the positive response to a diagnostic spinal tap had to be replicated. This was particularly important for patients with an impairment or tap-related improvement of only two of the symptoms characteristic for NPH. Moreover, our series included patients who preferred to have repetitive taps as compared to ventricular shunting. Thus, we were in the position to follow-up patients with suspected NPH over two or more spinal taps.We hypothesized that the patients with NPH and AD+ CSF changes will exhibit a different clinical response to a repeated spinal tap as compared with patients with NPH and AD− CSF.

## Materials and methods

### Study population

**Fig. 1:**
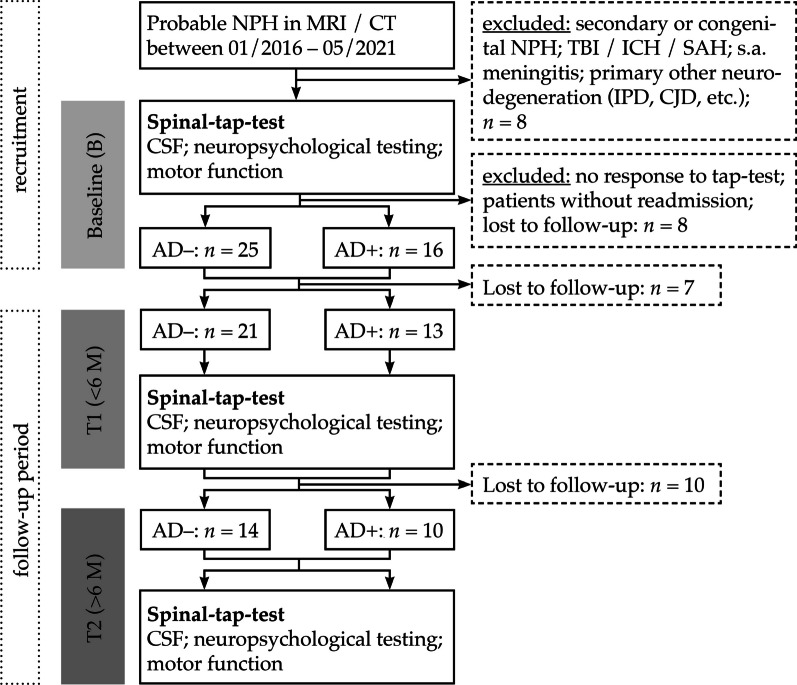
Selection-flow chart of NPH patients and dichotomization in AD+ and AD− group by CSF biomarker signature and typical neuropsychological features of Alzheimer disease. AD−: Alzheimer negative, AD+: Alzheimer positive. B: baseline visit (recruitment), T1: follow-up visit (< 6 month), T2: follow-up visit (> 6 month). NPU = neuropsychological testing, CT = cranial computed tomography, MRI = cranial Magnetic resonance imaging, TBI = traumatic brain injury, ICH = Intracranial hemorrhage, SAH = Subarachnoid hemorrhage, IPD = idiopathic Parkinson's disease, CJD = Creutzfeldt-Jakob disease, *n* = number

Forty-one consecutive patients between June 2015 and March 2021 were included into this study. The patients were of both sexes and admitted because of diagnostic work-up for suspected NPH according to international guidelines. All of them presented with typical clinical symptoms of NPH including cognitive deficits, a slurred, unsteady and slowed gait, and urine incontinence. Also, they had a communicating internal hydrocephalus on cranial MRI or cranial CT characteristic for NPH (Fig. 1).

According to the third edition of the International Guidelines the ventricular enlargement of lateral and third ventricles assessed in relation to the maximal inner skull diameter was associated with a patent Sylvian aqueduct and no macroscopic obstruction of CSF flow, there was a lack of cortical atrophy, presence of periventricular water content, and an increased callosal angle in the coronal plane [9]. Upon lumbar puncture the intracranial pressure was normal. Patients with suspected secondary NPH were excluded, e.g. NPH associated with a prior severe head trauma, meningitis or intracranial hemorrhage. In addition, patients with a suspected neurodegenerative disorder such as Parkinson’s disease or primary dementia were excluded (Fig. 1). Also, patients who underwent implantation of a ventricular shunt were excluded from this study. At the time of recruitment (Baseline point, B) we identified 41 patients with clinical symptoms and the morphologic aspect of NPH of which 25 were AD− and 16 AD+ in the CSF signature of biomarkers. We had patients with a first follow-up after 6 months (time point T1: 21 AD− patients, 13 AD+ patients). Also, there were patients with a second follow-up readmission longer than 6 months after the baseline study (time point T2: 14 AD− patients, 10 AD+ patients). The different points in time were summarized as Delta T. During each readmission for follow-up we executed the complete diagnostic spectrum according to our standardized protocol (spinal tap-test with CSF collection, gait analysis and neuropsychological testing) (Fig. 1).

### CSF collection and biomarker analysis

CSF samples of 40–50 ml were obtained by lumbar puncture at each time point (Baseline, T1, T2) after informed consent and processed as described elsewhere [17]. In short, quantitative analyses were performed by a commercial laboratory partner and classified according to standardized cut-off values (Additional file 1: Appendix Table A1) (MVZ Synlab Leverkusen, Germany). Standardized sandwich ELISA methods were used for measurement of the core biomarkers, namely the INNOTEST®-AMYLOID (1–42), INNOTEST® hTAU Ag, INNOTEST® PHOSPHO TAU (181P). In addition, the beta-amyloid (1–42)/(1–40) ratio was determined to eliminate preanalytical and material interference to achieve increased specificity of laboratory diagnosis for AD. Neuron-specific enolase and S100B were measured using the fully automated commercially available chemiluminescence immunoassays LIAISON® S100 and LIAISON® NSE (DiaSorin, Italy). For measurement of CSF protein, a photometric measurement technique was used.

### Gait and neuropsychological assessments

Patients were subjected to neuropsychological testing and a standardized testing of gait and mobility within 24 h before and 24–48 h after the spinal tap. Gait and mobility were assessed with (I) 10 Meter Walking Test (10-MWT; [18, 19]), and (II) Time-up-and-Go-Test (TUG; [20]). In addition, since there is a close association between cognition and gait [21] which has previously been studied in NPH using dual task conditions [22] the 10-MWT and TUG were administered also using dual task (DT) variants applying simple distractors, such as (IV) respectively counting backward with seven [23] or (IV.1) carrying a cup of water with the intent not to spoil the water (TUG-DT [24],). Gait was hand measured and the average of three attempts was used. The neuropsychological tests comprised (I) the clock drawing test (CDT, [25, 26]) to assess visual-spatial organization, (II, III) the German version of the verbal digit span and block tapping to assess auditory and visual memory span and working memory (WMS-R; BTf and BTr) [27], (IV) the mosaic test (MT, [28]) to assess visuoconstruction and alertness (intrinsic and phasic), (V, VI) figure copying (FC) and remembering (CERAD; dFrep) [29].

### Statistics

The baseline characteristics of demographic data were statistically evaluated using chi-squared test for sex, type 2 diabetes mellitus and urinary incontinence and the Mann–Whitney U test for all other comparisons (CSF biomarkers, gait values, neuropsychological results after standardization of all data via *z*-transformation). Changes in neuropsychological function and gait over time were analyzed by a repeated-measures ANOVA and post hoc Student paired t-test were calculated. Then a multiple linear regression was performed. In this exploratory study the level of significance chosen was 0.05, but differences of greater significance are stated specifically. Statistical analyses were performed using IBM SPSS Statistics for IOS version 27.0.1.0 (SPSS, Chicago, IL). The study was approved by the Ethics Committee of the Medical Faculty of the Heinrich-Heine-University Düsseldorf (#2019–390 and #2020–930).

## Results

### Demographic data

**Fig. 2:**
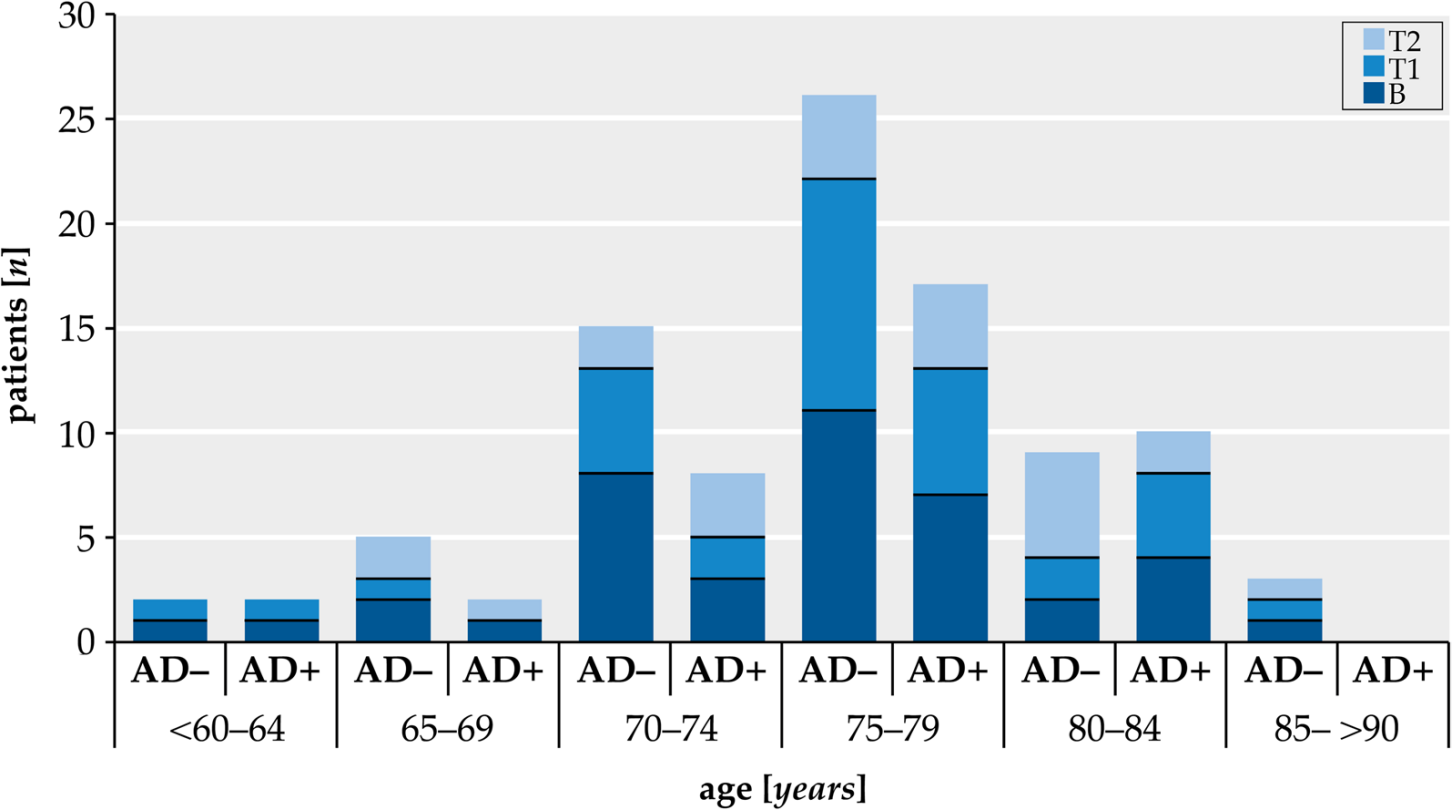
Age distribution of the patients. First admission (baseline = B), readmission (T1 ≤ 6 months) and second readmission (T2 ≥ 6 months). AD−: Alzheimer negative, AD+ : Alzheimer positive, *n* = number

The study cohort of 41 patients included the AD+ group (*n* = 26) with a CSF biomarker signature of AD and the AD– group (*n* = 25) with normal dementia biomarkers in CSF. The AD+ patients were at baseline on average 1.5 years older than the AD– patients (Table 1, Fig. 2). The age distribution between the two groups was different while there was no significant difference in age between the subgroups (T1: 75.9 y vs. 77.6 y, *p* = 0.246; T2: 77.3 y vs. 76.21 y, *p* = 0.516). However, the AD– group had a single peak between 70 and 80 years of age, the AD+ group had a preponderance of patients over 80 years (Additional file 1: Figure A1).

**Table 1:** Baseline characteristics of demographic data, motor and neuropsychological function at baseline (pre-tap)

Characteristic	AD− Patients	AD+ Patients	*p*-value
Number, n	25	16	
Females/males, n	14/11	6/10	0.248
Age, years	75.4 ± 4.9	76.9 ± 5.9	0.240
Type 2 Diabetes mellitus, n (%)	8 (32)	4 (25)	0.631
Urinary incontinence, n (%)	15 (60)	12 (75)	0.865
Intracranial pressure, cmH2O	18.3 ± 5.1	18.3 ± 3.8	0.758
Walking speed (10-MWT) (m/s)^a^	0.86 ± 0.26	0.83 ± 0.21	0.757
Time-up-and-go test (TUG) (s)^b^	15.07 ± 5.86	15.51 ± 5.57	0.788
Time-up-and-go dual test (TUG-DT) (s)	18.89 ± 9.94	20.0 ± 13.46	0.950
Clock drawing (CDT) (value)	2.69 ± 1.05	2.54 ± 1.14	0.765
Block tapping forward (BTf)^#^	−0.05 ± 1.03	−0.16 ± 0.77	0.932
Block tapping reverse (BTr)^#^	0.06 ± 0.9	0.05 ± 1.23	0.810
Mosaic test (MT)^#^	−0.19 ± 0.95	0.22 ± 1.15	0.381
Figure copying (FC)^#^	−0.45 ± 1.17	−0.77 ± 1.16	0.514
Delayed figure reproduction (dFrep)^#^	−0.91 ± 1.52	−1.85 ± 1.01	0.088

Values are mean ± SD. AD−: Alzheimer negative, AD+: Alzheimer positive. *n* = number

^#^*z*-value; CDT = clock drawing test, BTf = block tapping forward, BTr = block tapping reverse, MT = mosaic test, FC = figure copying, dFrep = delayed figure reproduction. Differences in the clinical data (sex, type 2 diabetes mellitus and urinary incontinence) were evaluated using Pearson’s chi-squared test. The other characteristics were compared using the Mann–Whitney U test. Statistical significance in group comparison was considered to be present at *p* < 0.05

^a^Age-related walking speed: 2.1 ± 0.35 m/s

^b^Age-related normal range: 8.2–10.2 s,

### Gait functions

**Fig. 3:**
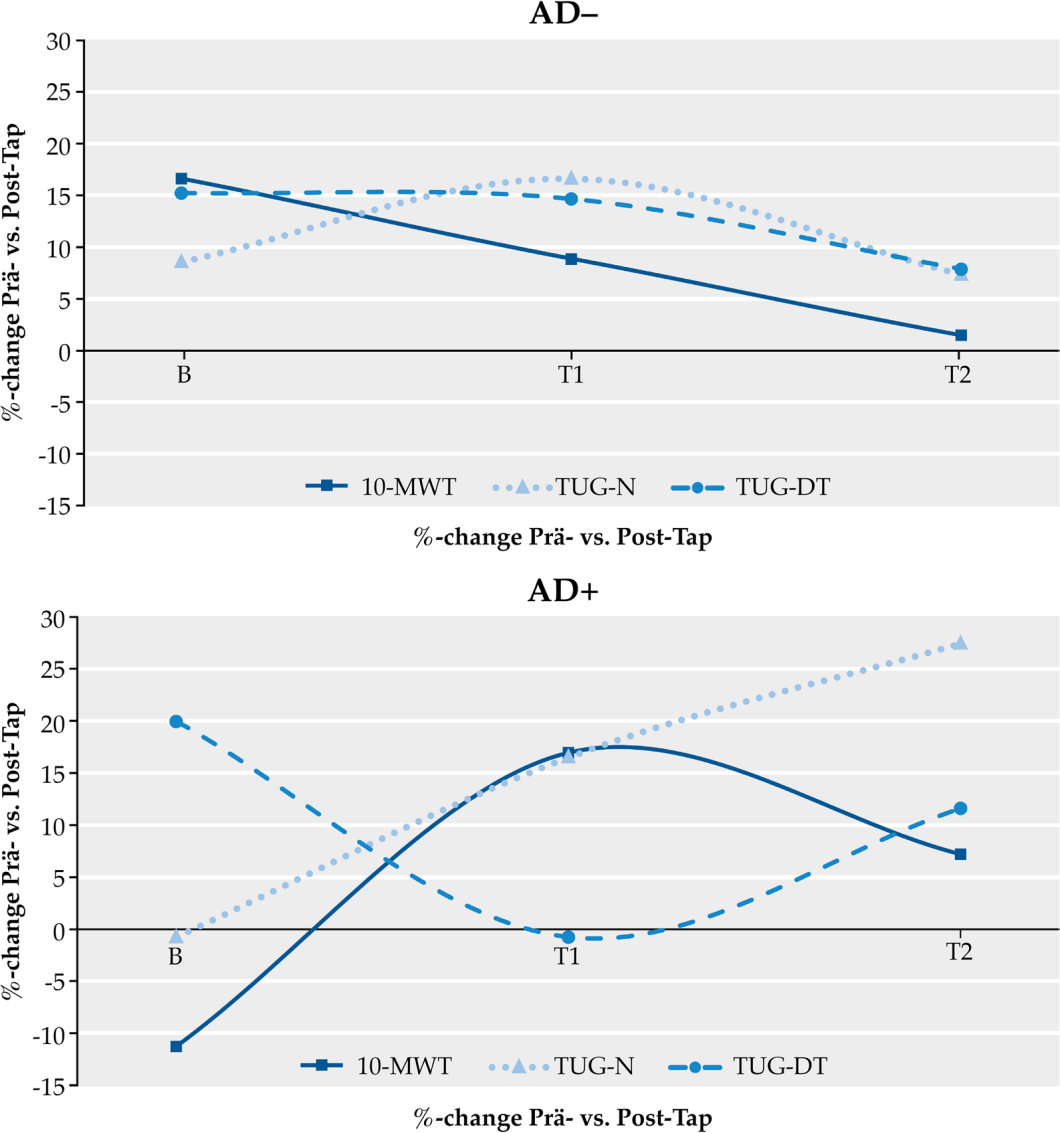
Temporal dynamics of gait functions as %-change of pre-tap and post-tap results for each time point of investigation. AD−: Alzheimer negative, AD+: Alzheimer positive. B: baseline visit (recruitment), T1: follow-up visit (< 6 month), T2: follow-up visit (> 6 month). 10 m walk test (10-MWT), time-up-and-go test (TUG-N), time-up-and-go dual test (TUG-DT)

In both groups walking speed was severely decreased to half of the age-range and the Time-up-and-Go-Test was severely slowed at baseline (Table 1). After the spinal tap walking speed was improved in the AD+ patients. But this effect tapered over time (Fig. 3). In comparison, the spinal tap tended to be increasingly effective in the AD+ patients as evident from the 10 m walking test and the Time-up-and go Tests.

### Neuropsychological testing

**Fig. 4:**
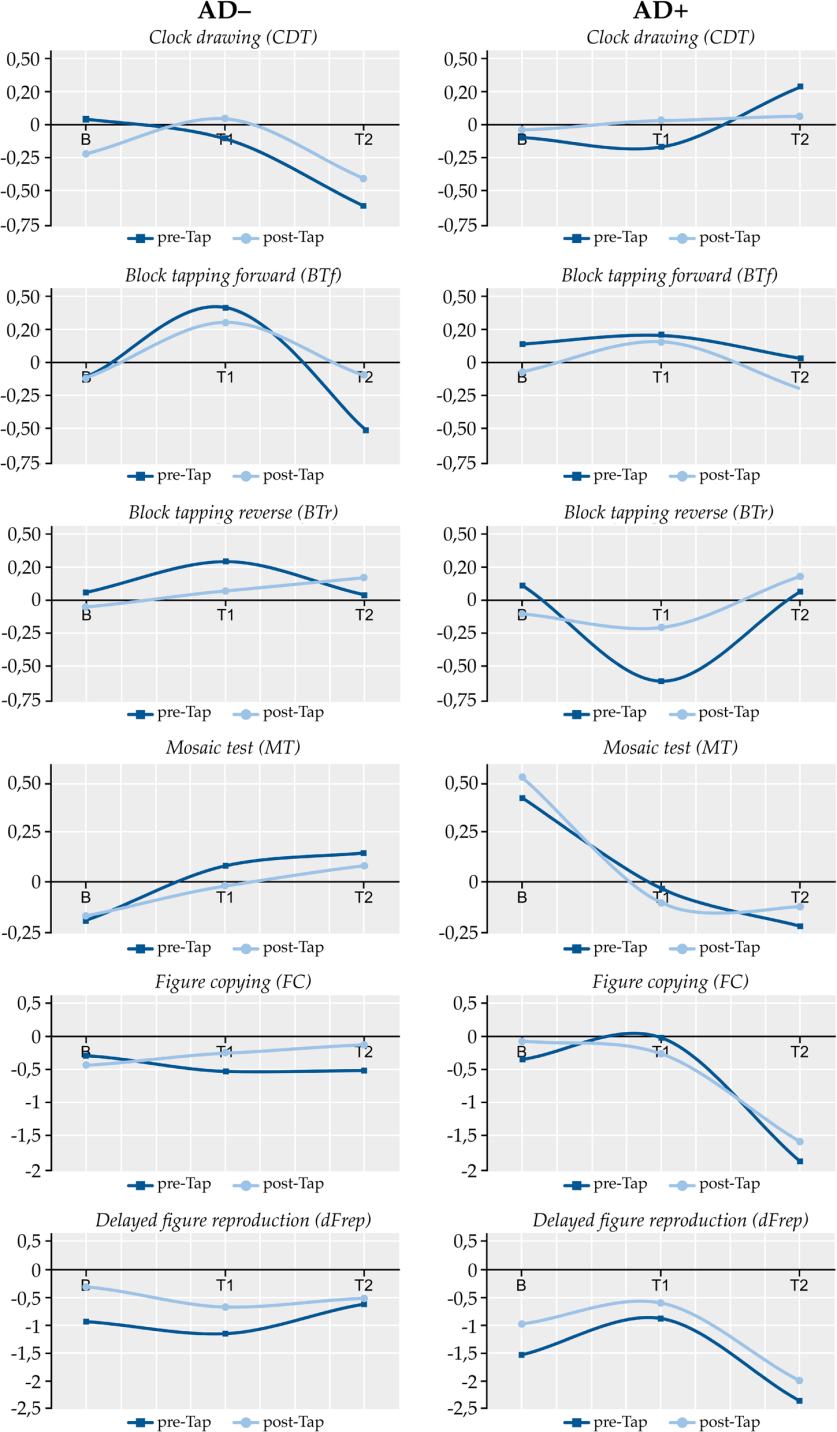
Temporal dynamics of the neuropsychological test results for each time point of visits. The *z*-transformed mean values and the 2nd order polynomial interpolations are shown. AD−: Alzheimer negative, AD+: Alzheimer positive. B: baseline visit (recruitment), T1: follow-up visit (< 6 month), T2: follow-up visit (> 6 month). CDT = clock drawing test, BTf = block tapping forward, BTr = block tapping reverse, MT = mosaic test, FC = figure copying, dFrep = delayed figure reproduction

The neuropsychological results were impaired in both patient groups at baseline but there were no group differences (Table 1). The profiles of the neuropsychological functions were quite different among the two patient groups and revealed different dynamic patterns over time (Fig. 4). Most importantly, clock drawing deteriorated in the AD− patients, whereas block tapping, visuoconstruction and figure copying and reproduction remained stable. Moreover, in the AD− patients figure copying and delayed figure reproduction benefitted from the spinal tap initially, while this effect vanished over time. In contrast, visuoconstruction and figure copying declined over time in the AD+ patients. Importantly, however, block tapping was improved in the AD+ patients after the spinal tap even on the follow-up time points (Fig. 4).

### Urine incontinence

At the time of baseline (B), 60% of the AD–patients had incontinence, in the group of AD+ patients the proportion was 75%. The patients reported an improvement of urine incontinence after the spinal tap. Upon follow-up, however, there was no significant difference between the groups (B: *p* = 0.329; T1: *p* = 0.660; T2: *p* = 0.735;) (Additional file 1: Figure A2).

### CSF biomarkers

**Fig. 5:**
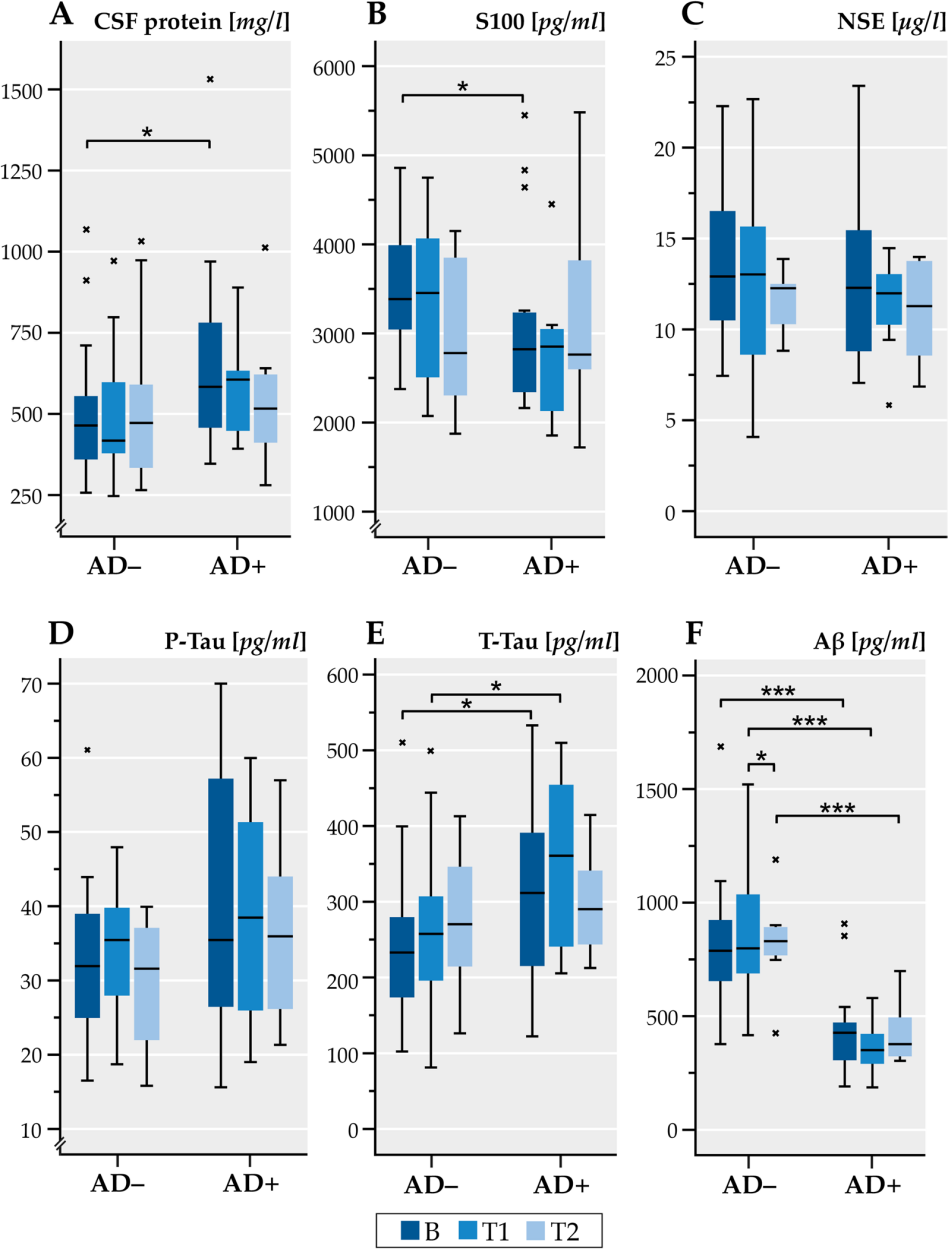
CSF biomarker concentrations in the patients of CSF protein (**A**), S100 (**B**), NSE (**C**), Phospho-Tau (**D**), Tau protein (**E**) and β-Amyloid 1–42 (**F**) compared between AD− and AD+. Horizontal lines within boxes show median values, boxes show upper and lower interquartile ranges, whiskers indicate the 95% confidence interval, a cross (x) in the figure indicate outlier values. AD−: Alzheimer negative, AD+ : Alzheimer positive. B: baseline visit (recruitment), T1: follow-up visit (< 6 month), T2: follow-up visit (> 6 month). T2: study visit more than 6 month (> 6 M.). S100: S100 protein; NSE: neuron-specific enolase; T-Tau: total Tau; P-Tau: Tau phosphorylated at threonine 181; Aβ42: Amyloid-β 1–42 protein. Differences analyzed with Mann Whitney U test. *p < 0.05, **p < 0.01, ***p < 0.001

**Fig. 6:**
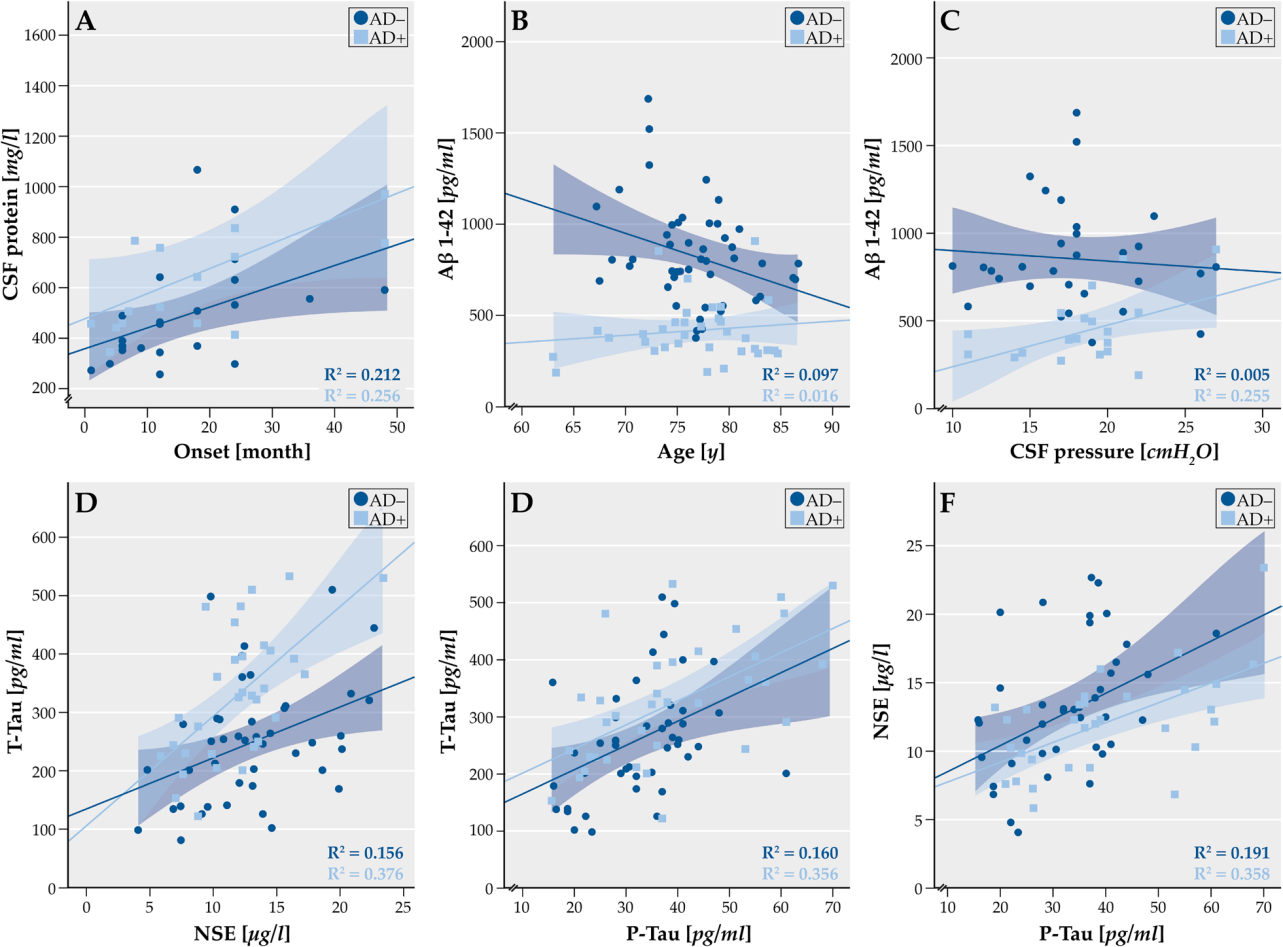
Correlation analysis. CSF protein versus onset (**A**), Aβ 1–42 versus age (**B**), T-Tau versus NSE (**C**), Aβ 1–42 versus CSF pressure (**D**), T-Tau versus P-Tau (**E**) and NSE versus P-Tau (**F**) in Aß– and Aß+ patients. Pearson R2 values and significance level were calculated, and linear graphs adjusted according to the values. All time points of B, T1 and T2 are included in correlation analysis. AD−: Alzheimer negative, AD+ : Alzheimer positive. B: baseline visit (recruitment), T1: follow-up visit (< 6 month), T2: follow-up visit (> 6 month). Onset: Time since symptom onset; NSE: neuron-specific enolase; T-Tau: total Tau; P-Tau: Tau phosphorylated at threonine 181; Aβ42: Amyloid-β 1–42 protein

The CSF protein content was elevated at baseline in the AD + patients which was significantly higher than in the AD– patients (Fig. 5a). This difference decreased upon follow up. The S100 protein was elevated in the AD− patients and particularly higher than in the AD+ patients at baseline (Fig. 5b). The T-tau protein was slightly elevated in both patient groups but particularly in the AD+ patients (Fig. 5e, Additional file 1: Table A1). Most importantly, Aß-protein was profoundly decreased (*p* < 0.001) in the AD+ patients with lower values as in the AD− patients (Fig. 5f). Quite differently, NSE and P-Tau showed a wide range of variation in both patient groups with no differences among them (Fig. 5c, d, Additional file 1: Table A1). Figure 6a shows that the CSF protein content increased in both patient groups over the follow-up period. This relation was significant. There was a different relation of the Aß-protein in CSF with the age of the patients. While the Aß-levels were stable over the observation period in the AD+ patients, they declined in an age-related fashion in the AD− patients (Fig. 6b). Moreover, the CSF-pressure increased in relation to the Aß-protein in the AD+ patients but tended in decrease in the AD− patients (Fig. 6c). The CSF pressure was related in the AD+ patients with the NSE and Aß levels and with the P-tau level in the AD− patients. Furthermore, it is shown that the T-tau level was related to the NSE level and P-tau level as was the NSE-level to the p-tau level in both patient groups (Fig. 6d–f). This relation was significant in the AD+ patients for NSE and P-tau and T-tau, but for NSE and Aß, P-tau and T-tau as well as for Aß and P-tau and T-tau in the AD– patients. The relation of P-tau and T-tau was significant in both patient groups. Both groups also differed with respect to the beta-amyloid (1–42)/(1–40) ratio (*p* = 0.003), which showed an abnormally lower ratio (0.69 (mean) ± 0.24 (SD)) in the AD+ patients at the first spinal tap.

## Discussion

In this study, we followed 41 consecutive NPH patients on repeated admissions concerning the effect of a spinal tap on gait and neurocognitive functions, urine incontinence and neurodegenerative biomarkers in CSF over up to 49 months. Two distinct patient subgroups were identified based on the biomarkers in CSF. The AD+ patients had CSF biomarkers suggestive of dementia of the Alzheimer type, while the AD− group exhibited no such changes in CSF. Most importantly, the AD+ patients continued to benefit concerning gait from repeated spinal taps, while this effect vanished in the AD– groups. Neuropsychological testing revealed a heterogeneous response to the spinal tap in these patients. In the AD− patients clock drawing deteriorated progressively, while visuomotor coordination as evident from figure copying and reproduction were improved after the first spinal tap but vanished on follow-up. For comparison, visuoconstruction and figure copying declined in the AD+ patients, whereas they continued to improve in block tapping after the spinal tap on follow-up. To the best of our knowledge this is the first follow-up study to provide such a longitudinal comparison of the effect of the spinal tap in patients with NPH with either AD+ or AD− biomarker signatures in CSF.

Our results suggest a coincidence of Alzheimer disease in a subgroup of patients presenting with NPH. Accordingly, there seems to be a neurodegenerative disorder leading to cognitive decline and an associated disturbance of CSF re-uptake [30, 31]. Conversely, patients with Alzheimer disease typically do not suffer from NPH. For example, it was found in a randomized controlled study in patients with Alzheimer’ disease without radiographic changes suggestive of NPH that a ventriculo-peritoneal shunt to clear macromolecules from CSF did not delay the disease progression [32]. On the first glance it appears to be counterintuitive that patients with NPH and AD+ constellation improve upon CSF drainage. But recently it was found that the positive effect of a spinal tap was related to amyloid PET changes in the brain [33]. Furthermore, patients with biomarkers of Alzheimer disease showed a cognitive decline, although they were improved for about three months after the spinal tap [34]. As we were able to show in a previous study [15], the CSF parameters also had a positive effect in the spinal tap in patients with Alzheimer signature. These results are in line with studies that have shown similar effects, including NPH patients with amyloid present in brain biopsies [35].

The relation of abnormal levels of these biomarkers in CSF to the clinical relevance of these proteins in cerebral cortex is not well understood as yet, although they come into focus for the differentiation of degenerative disorders including NPH and their mimics [36]. S100 and NSE were elevated in the AD+ and AD− patients as they are markers of astrocytic and neuronal damage as well as of the integrity of the blood brain barrier. Recently, it was shown that ß-amyloid and Tau-protein are deposited in cerebral cortex in relation to ageing with the consequence of a memory decline [37]. Moreover, beta-amyloid and Tau loading in cerebral cortex effectively modulates the deficit patterns in patients with microvascular brain degeneration and in Lewy body disorders [38, 39]. In accordance with previous evidence [40] the majority of our patients had marked changes in the cerebral white matter. We observed moderate and severe confluent changes as classified by Fazekas [41] in the periventricular and deep hemispheric white matter in both the AD+ and AD− patients. As this was not the primary goal of this study, we have initiated a subsequent study to assess these changes in NPH patients in a statistical fashion. Moreover, there also seems to be an overlap of NPH and beta-amyloid load in brain tissue [42, 43]. Interestingly, in a prospective study of 52 NPH patients memory improvement after the tap was associated with beta-amyloid and Tau pathology on brain biopsy and lower ß-amyloid and higher Tau in CSF [44]. In fact, in a large meta-analysis of 25 studies with over 664 patients it was found that patients with NPH had similar patterns of beta-amyloid and Tau in CSF as those with Alzheimer disease [45]. Notably, patients with NPH and AD+ pattern in CSF were reported not to show an increase of cerebral blood flow after shunting in contrast to patients with NPH and an AD- pattern [46].

The CSF pressure seems to be of particular importance in explaining the NPH disease and the patient dichotomy. Based on the findings of the CSF-biomarkers in this study we would like to suggest that changes in intracranial and intraventricular pressure gradients may lead to increased drainage resistance and, consequently, to a reduced clearance of toxic metabolites of the brain. It is likely that the clearance function of the toxic metabolites, which is disturbed in patients with idiopathic and neurodegenerative NPH, is partially compensated by the CSF puncture. It should be emphasized, however, that due to the relatively low number of patients included in either patient group in this exploratory study definite conclusions have to await confirmation by larger studies.

Finally, a shunt does not seem to raise the cerebral blood flow in NPH patients with findings in CSF typical for Alzheimer disease as much as in NPH patients without such CSF abnormalities [47]. These data are corroborated and extended by our findings that suggest an association of changes in cognition and gait after CSF drainage and Alzheimer disease as a consequence of a neurodegenerative disease affecting cognitive functions, gait and urine continence as well as CSF drainage. Since this study is not suited to exclude that there may be AD− patients who benefit from a spinal tap, we speculate that NPH of neurodegenerative cause is more frequent than a truly idiopathic NPH. While an idiopathic NPH without degenerative CSF changes is supposed to be the appropriate candidate for a shunt, a sustained improvement upon spinal drainage over three years was reported to be rare [6]. Our CSF data and statistical analysis suggest a coincidence of dementia of the Alzheimer type in a subgroup of patients presenting with NPH. Furthermore, the postulated dichotomy between neurodegenerative and idiopathic NPH could also be confirmed in this longitudinal study. Nevertheless, our findings suggest that patients who initially have no pathological CSF findings develop an Alzheimer signature over time. Here we confirmed our prior observation [15] that repeated lumbar punctures improve both motor and cognitive deficits, which show regressive after removal of cerebrospinal fluid in patients with NPH that are AD+. The pathophysiological cause of NPH is not well understood. There is, however, recent evidence from an experimental MRI study showing that the intrathecally injected MRI contrast agent is cleared from CSF with delay in patients with NPH as compared with a control sample [31, 32]. These data suggest an impairment of the glymphatic pathway which is assumed to be located in meningeal lymphatic vessels. Moreover, it was shown that the glymphatic pathway declines in normal aging. Consequently, the impairment of the glympathic pathway in NPH appears to exceed that of normal aging and may occur as second pathology in patients with Alzheimer disease. In our Centre we consider ventricular shunting as indicated only if two complete investigations at least three months apart from each other reveal a significant improvement in cognitive, motor and urine control functions as reported in this paper. Therefore, a study is under way in which we assess in our patient population how the patients fare after shunting.

## Conclusion

The longitudinal follow-up showed that NPH patients with and without AD constellation in CSF had a different response to the spinal tap. Both groups differed in terms of motor and neurocognitive functions and reduction of incontinence. The CSF biomarkers determined, in particular total protein, Aβ 1–42, T-tau and NSE, appear to represent a significant pathophysiological clue. With increasing age, the Aβ 1–42 values in the initially AD− patients decreased to a pathological level. The patients with initially pathologically low Aβ 1–42 values showed a positive response to the spinal tap in the long term. Thus, this study supports the previous findings of different comorbidities within the spectrum of patients with NPH.

### Supplementary Information

**Additional file 1**: Graphical illustration of subgroup-specific and sex-independent age distribution, change in incontinence over time, and time courses of CSF biomarkers.

## Data Availability

The data presented in this study are available upon request from the corresponding author. The data are not publicly available for reasons of medical law and data protection.
