# Supplementary material for: Evolution of neurodegeneration in patients with normal pressure hydrocephalus: a monocentric follow up study
Source: Neurol Res Pract. 2023 Sep 7;5:52. doi: 10.1186/s42466-023-00272-6 (PMC10483764; doi:10.1186/s42466-023-00272-6)
Supplement: Supplementary file 1 — Additional file 1: Graphical illustration of subgroup-specific and sex-independent age distribution, change in incontinence over time, and time courses of CSF biomarkers. [file 42466_2023_272_MOESM1_ESM.docx]

**Appendix A**


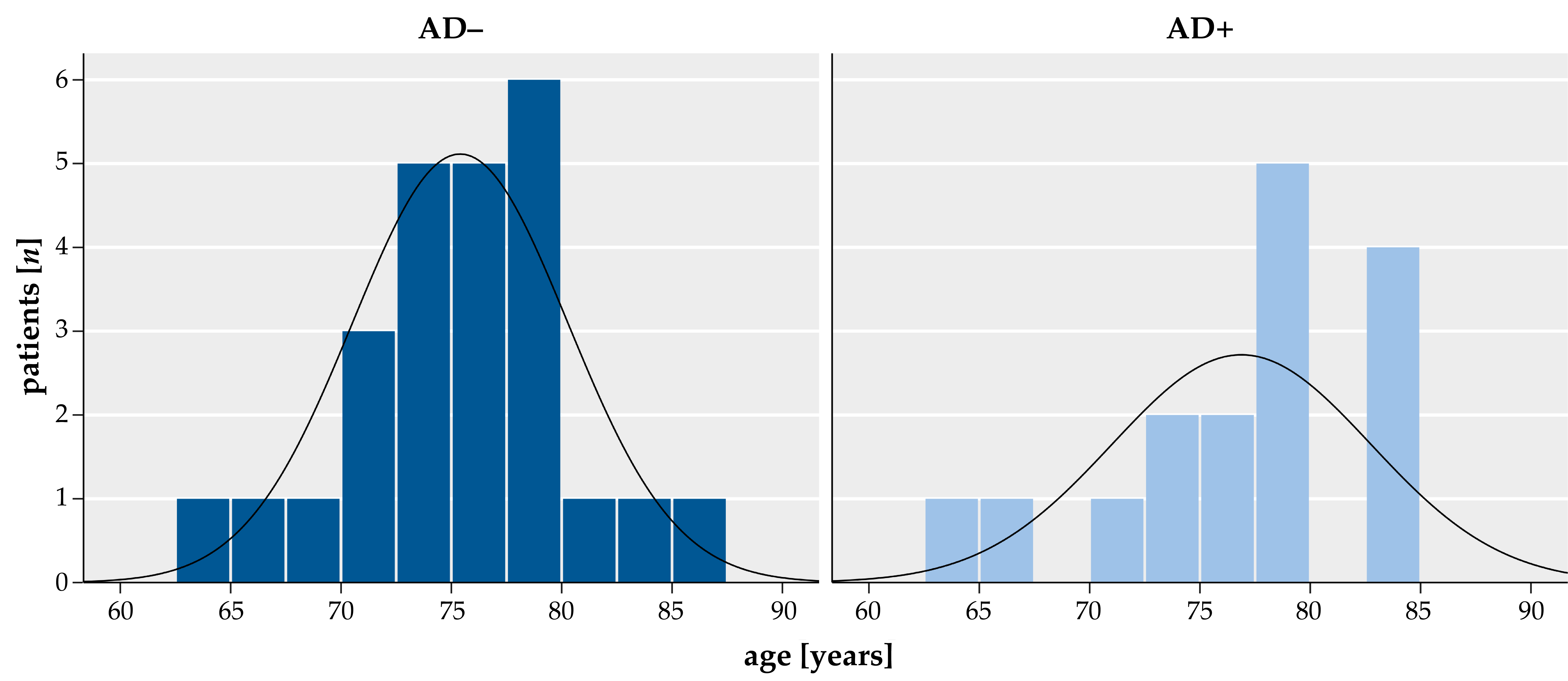


**Figure A1.** *Subgroup-specific and gender-independent age distribution for the baseline visit of the follow up.* AD–: Alzheimer negative, AD+: Alzheimer positive, *n* = number.


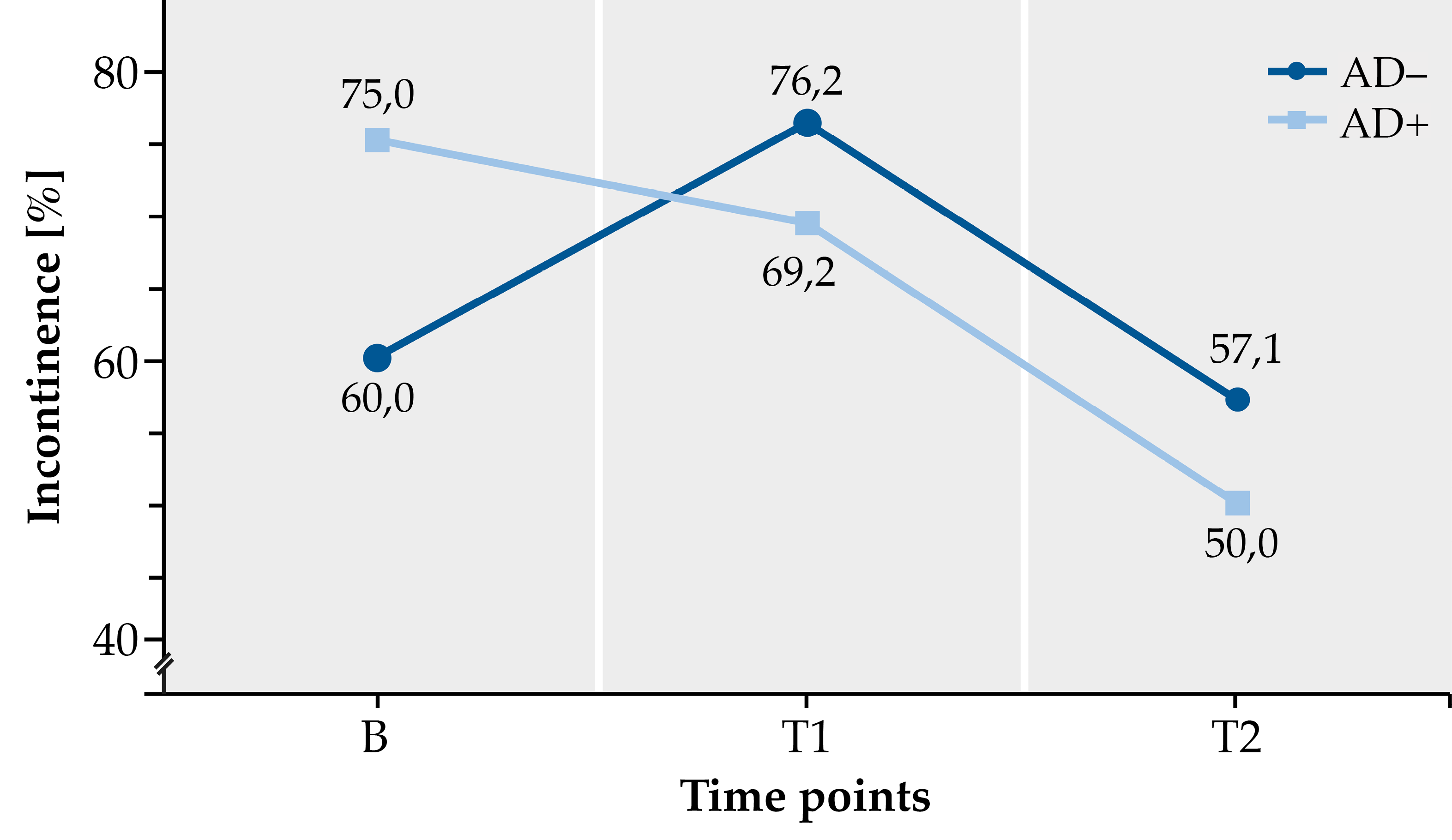


**Figure A2.** *Temporal dynamics of incontinence.* The percentage changes in the mean values are given in [%]. AD–: Alzheimer negative, AD+: Alzheimer positive. B: baseline visit (recruitment), T1: follow-up visit (< 6 month), T2: follow-up visit (> 6 month).

**Table A1.** Levels of Cerebrospinal fluid biomarker of the study population over time. Patients grouping of AD– and AD+ based on CSF biomarkers in the timescale of B, T1 and T2.

| **Parameter** | **AD– Patients** | | | **AD+ Patients** | | | **Normal range** |
| --- | --- | --- | --- | --- | --- | --- | --- |
|  | **B** | **T1** | **T2** | **B** | **T1** | **T2** |  |
| S100 (pg/ml) | 3579  ± 679,2 | 3371,3  ± 895,8 | 2988,8  ± 858,2 | 3157,0  ± 1046,7 | 2731,3  ± 795,3 | 3258,0  ± 1352,6 | < 2700 |
| NSE (µg/l) | 13,4  ± 4,3 | 12,6  ± 5,6 | 12,6  ± 3,5 | 12,7  ± 4,5 | 11,4  ± 2,6 | 11,0  ± 2,9 | < 13,0 |
| P-Tau (pg/ml) | 32,8  ± 10,6 | 34,0  ± 8,5 | 29,7  ± 9,5 | 40,0  ± 17,3 | 38,1  ± 14,5 | 36,6  ± 12,6 | < 61 |
| T-Tau (pg/ml) | 240,4  ± 97,0 | 258,7  ± 114,2 | 275,4  ± 94,5 | 319,5  ± 125,4 | 356,8  ± 109,6 | 297,4  ± 69,2 | < 290 (normal) |
|  |  |  |  |  |  |  | 291-452 (borderline) |
|  |  |  |  |  |  |  | > 453 (pathological) |
| Aβ 1-42 (pg/ml) | 801,7  ± 265,3 | 879,2  ± 310,5 | 824,2  ± 211,5 | 439,7  ± 199,8 | 372,9  ± 115,4 | 424,8  ± 133,0 | > 650 (normal) |
|  |  |  |  |  |  |  | 550-650 (borderline) |
|  |  |  |  |  |  |  | < 550 (pathological) |
| CSF protein (mg/l) | 490,3  ± 194,5 | 493,7  ± 185,7 | 523,9  ± 258,9 | 664,6  ± 294,4 | 573,9  ± 148,1 | 549,0  ± 220,2 | 150-450 |

Values of S100, NSE, Phospho-Tau, Tau protein, β-Amyloid 1-42, CSF protein and CSF pressure are presented as means for each collection time-point ± SD. AD–: Alzheimer negative, AD+: Alzheimer positive. B: baseline visit (recruitment), T1: follow-up visit (< 6 month), T2: follow-up visit (> 6 month). S100: S100 protein; NSE: neuron-specific enolase; T-Tau: total Tau; P-Tau: Tau phosphorylated at threonine 181; Aβ42: Amyloid-β 1-42 protein.
